# Increasing roughage quality by using alfalfa hay as a substitute for concentrate mitigates CH_4_ emissions and urinary N and ammonia excretion from dry ewes

**DOI:** 10.1111/jpn.13223

**Published:** 2019-10-09

**Authors:** Chunmei Wang, Cheng Zhang, Tianhai Yan, Shenghua Chang, Wanhe Zhu, Metha Wanapat, Fujiang Hou

**Affiliations:** ^1^ State Key Laboratory of Grassland Agro‐Ecosystems Key Laboratory of Grassland Livestock Industry Innovation Ministry of Agriculture and Rural Affairs College of Pastoral Agriculture Science and Technology Lanzhou University Lanzhou China; ^2^ Agri‐Food and Biosciences Institute Hillsborough UK; ^3^ Faculty of Agriculture Department of Animal Science Tropical Feed Resources Research and Development Center (TROFREC) Khon Kaen University Khon Kaen Thailand

**Keywords:** alfalfa hay, concentrate supplementation, methane emissions, N utilisation, nutrient digestibility

## Abstract

Twelve Hu sheep × thin‐tail Han crossbred dry ewes with an average body weight of 32.6 ± 0.68 kg and an age of 3 years were arranged in a 3 × 3 Latin square design, with each experimental period of 24 d to evaluate the effect of substituting alfalfa hay in a portion of concentrate on nutrient intake, digestibility, N utilisation efficiency and methane emissions. The ratios of corn straw to alfalfa to concentrate for 3 diet treatments were 60:0:40, 60:15:25 and 60:30:10, respectively. Intake and digestibility were measured for each of the ewes, which were housed in individual metabolism crates for 6 d after an adaptation period of 14 d, and the feed was offered at 1.2 ME_m_ to ensure approximately 10% orts. Methane emissions were determined in a respiration chamber for 2 consecutive d. An increase in the levels of alfalfa as a substitute for concentrate significantly increased the roughage, NSC and ADF intake and faecal N output as a proportion of N intake and manure N output. Furthermore, this increase in alfalfa input levels decreased DE, ME and N intake; nutrient digestibility; DE/GE, ME/GE and CH_4_ emissions per day; CH_4_ output expressed as a portion of the DM, OM and GE intake; and urinary N and ammonia N output, especially between extreme treatments. Alfalfa input levels had no effect on the BW, DM and GE intake; the EB or EB/GE intake; and the retained N. This study indicated that increasing alfalfa input as a substitute for concentrate could significantly decrease the digestibility, CH_4_ emissions and urinary N and NH_4_^+^‐N outputs; and shift the N excretion from urine to faeces; and could sustain a similar DM intake.

## 1 INTRODUCTION

The use of crop straw as roughage feed has a long history, especially in arid–semiarid areas, which occupy more than 40% of the terrestrial area in which agriculture land is mainly used for grain crops. For straw used as feed, more than 50% of this portion is directly chopped into small pieces (MOA, 2011), and then offered to animals together with grain concentrate in China. Since 2003, grazing exclusion and grassland restoration implemented for ecological protection and restoration have resulted in ruminant production systems becoming more dependent on mixed diets of straw and concentrate as the numbers of ruminants housed in pens for intensive production have increased. Straw as a low‐cost roughage with high lignin, low crude protein and low minerals cannot meet the nutrient requirements of ruminants and would increase CH_4_ emissions. Studies have shown that the problem of low microbial protein yield with poor‐quality roughage‐based diets cannot be simply solved or completely compensated by supplementation with high amounts of concentrates (Pathak, 2008), and high concentrate input also increases the risk of metabolic disorders (Tayyab, Wilkinson, Charlton, Reynolds, & Sinclair, 2019). Increasing the forage quality could improve concentrate‐sparing effect (Keady & Hanrahan, 2015), and the response in improving intake and digestibility from concentrate input could be overridden by high‐quality forage (Ramos, Tejido, Martínez, Ranilla, & Carro, 2009; Wang et al., 2019). Meanwhile, steadily increasing the demand for milk and meat much coming from ruminants would increase the proportion of food used as feed against the background of limited available arable land and further exacerbation of global climate change, with more than 13.3% of the world's cereal grains being offered to ruminants (Eisler et al., 2014) and with this proportion increasing in areas such as Brazil (Carvalho et al., 2019) and China. The increased demand for grain in feedlots is increasing the demand for agricultural lands, thereby adding pressure to the conversion of native ecosystems to agriculture, with further considerable environmental impacts at regional and global scales (McAlpine, Etter, Fearnside, Seabrook, & Laurance, 2009). These facts reveal the need to explore an alternative management where a substitute is provided for a portion of concentrate input to reduce grain supplementation as feed.

Alfalfa has an absolutely important role in ruminant production, especially for dairy cows (Wang, 2010). As a low‐cost protein feed, alfalfa is characterised by high water utilisation efficiency and yield (Sen, Makkar, & Becker, 1998), tends to be rich in the major minerals and certain trace elements (McDonald et al., 2011) and could offer sufficient protein to avoid the incidents of animal diseases caused by unbalanced nutrients (Huang, Mo, & Zhou, 2005), especially in developing countries, and in hot and/or arid–semiarid areas for small households, which account for much of the increase in sheep and goats numbers (Morgavi, Eugene, Martin, & Doreau, 2011). Alfalfa widely planted in the mixed crop‐livestock zone of Gansu Province accounts for more than 30% of total alfalfa area in China and could be used as a self‐sufficient substitute for expensive concentrate. Meanwhile, this area of alfalfa is increasingly encouraged by this move to “change grain to forage.” Hence, feed shifting from only a mixture of straw and concentrate towards more alfalfa input to replace a portion of concentrate is probably an alternative strategy to improve roughage quality and save cost.

Previous studies focused mainly on the effects of alfalfa input levels and/or the concentrates replaced by alfalfa on productive performance and milk quality (Baldwin, Kesler, & Hargrove, 1983; Kobayashi et al., 2017). Furthermore, alfalfa containing secondary metabolites has the potential to reduce methane emissions (Morgavi et al., 2011) without negative or even positive effects on ADG for calves (Kobayashi et al., 2017). Researches into nutrient digestibility, N utilisation and CH_4_ emissions focus less on sheep than on other ruminant species. The optimal alfalfa input level, when used as a substitute for a portion of concentrate for ewes, is not well defined for balanced digestible energy and protein to reduce CH_4_ emissions without adverse effects on nutrient intake. Hu × thin‐tail Han crossbred sheep, a major breed kept in Linze County, typically provided a mixture of 60% maize straw and 40% concentrate according to prevailing local management, were used as experimental animals in a benchmark diet treatment. The objective was to evaluate the effects of alfalfa as a substitute for a portion of concentrate for ewes on nutrient intake and digestibility, N utilisation and CH_4_ emissions.

## 2 MATERIAL AND METHODS

### 2.1 Experimental design and diets

Twelve Hu sheep × thin‐tail Han crossbred dry ewes (non‐lactating and non‐pregnant), aged 3 years and weighing 32.6 ± 0.83 kg, were arranged in a 3 × 3 Latin square design experiment, with a portion of their concentrate being replaced with 3 different levels of alfalfa hay in 3 experimental periods. In each period, 12 ewes were divided into 2 groups (2 ewes per alfalfa replacement level, *n* = 6 per group). Each group of 6 ewes was housed in 6 individual pens for 14 d, and then transferred to individual crates (1 sheep/1 crate) and remained for 7 d with feed intake and faeces and urine outputs measured during the final 6 d. Finally, the 6 sheep were moved to individual indirect open‐circuit respiration chambers (one ewe per crate per chamber) for 3 d with feed intake and faeces and urine outputs, O_2_ consumption and CO_2_ and CH_4_ production measured during the final 2 d. Therefore, a 3‐d interval existed between the first and second groups in the experiments. Each metabolism crate contained a roughage feed bin, a concentrate feed box, a drinking water container and separate trays for collecting faeces and urine. Sheep were provided free access to water throughout the experimental period.

Feed was offered at 1.2 maintenance of metabolic energy requirement (ME_m_) to ensure approximately 10% orts, and the ME_m_ was as recommended by Feeding Standard of Meat‐producing Sheep and Goats, China (Zhang, 2010). Well‐balanced digestible energy and protein for the diet treatments were formulated by changing the ingredients of concentrates (Table 1). The mixture of chopped alfalfa and corn straw (3–5 cm in length) was offered as two equal portions at 1,000 and 1,700, respectively, and pelleted concentrate was offered at 1,330 daily. Residual feed was collected and weighed before the morning feeding. Diet treatments were designed based on a constant 60% corn straw DM, so that alfalfa hay was offered as a substitute for a portion of the concentrate in the following proportions: 0:40, 15:25 and 30:10 (AH0, AH15 and AH30), respectively. The actual ratios of corn straw to alfalfa hay to concentrate were 57.6:0:42.4, 58.6:14.7:26.7 and 59.2:29.4:11.4. Corn straw harvested from seed production corn was purchased from local farmers at harvesting in October. Perennial alfalfa (Golden Empress cultivar) cultivated in 2010 in Linze Grassland Ecological Experiment Station of Arid Area, situated at 39.24°N, 100.06°E, was harvested at the beginning of flowering at the first growth stage in 2016. Concentrates containing wheat bran, corn, soybean meal and mineral and vitamin premix were purchased at the local market. The ingredients and nutrient values for the diet treatments are presented in Table 1.

**Table 1:** Ingredient and nutrient value for diet treatments (g/kg DM or MJ/kg DM)

Ingredient	AH0	AH15	AH30	Nutrient	AH0	AH15	AH30
Alfalfa	0	150	300	DM	923	925	928
Maize straw	600	600	600	OM	904	900	893
Wheat bran	284	135	0.10	NDF	599	595	595
Soybean meal	109	105	94.8	ADF	321	356	393
Maize	2.0	5.0	0.10	EE	24.7	20.6	16.7
Mineral premix	4.0	4.0	4.0	N	21.8	20.6	20.3
Vitamin premix	1.0	1.0	1.0	GE	18.1	17.8	17.6
				DE	10.4	10.3	10.2

Note

Mineral premix (Zhenjiang Tianhe Biotechnology) contains 2 g Cu, 20 g Fe, 12 g Zn, 15 g Mn, 0.15 g I, 0.1 g Se and 300 g Ca per kg DM.

Vitamin premix (Shandong Shenlong Animal Health Products) contains 1,102.3 × 10^4^ IU VA, 165.3 IU VD3, 5,512 IU VE, 4,409 mg VK, 551 mg VB1, 1,102 mg VB2, 7,560 mg VC, 8,112 mg folic acid, 7,560 mg L‐pantothenic acid, 10 g K^+^ and 7.5 g Na^+^ per kg DM.

DE concentration was calculated by the equation recommended by Zhang (2010).

### 2.2 Measurements

#### 2.2.1 Sample collection

The body weight for each ewe was determined before the adaptation period, before the ewe was transferred in and after the ewe was removed from the metabolism crate and chamber. Roughage and concentrate offered and refused were weighed for each ewe and sampled daily to measure the DM content at 65°C for 48 hr during each experimental period. Samples of offered roughage and concentrate were combined once weekly and then milled through a 1.0 mm pore sieve for determination of gross energy (GE), ash, nitrogen (N), neutral detergent fibre (NDF), acid detergent fibre (ADF), non‐structural carbohydrate (NSC), ether extract (EE) and phosphorus (P) concentrations. Residues of feed were bulked for each ewe during each experiment period, and the chemical compositions were analysed.

During each period of the digestibility trials, faeces and urine (20 ml of 30% sulphuric acid was added to each urine collection tray) outputs from each ewe were measured daily. Faeces and 20% of the urine excreted were stored in a 4°C freezer for the first 5 d. After the last day of collection, faeces and urine were thoroughly admixed, and then a representative sample was taken. Each fresh faeces sample was divided into two subsamples. One portion was used for analysing N content on a fresh basis, and another portion was used for measuring DM content at 65°C for 96 hr and then forced through a 1.0 mm sieve for analysing the GE, ash, NDF, ADF, EE and P contents on a DM basis. Urinary samples were used to determine N, P and GE concentrations, and GE concentration was measured using 10 ml oven‐dried samples contained in filter paper, and the filter paper was with known weight and energy concentration.

Feed offered and refused, and oven‐dried faeces samples on a DM basis were stored at 4°C, and fresh faeces and urine samples were stored at −20°C until laboratory analysis.

#### 2.2.2 Chemical analysis

Dry matter content was measured in a forced drying oven at 65°C (DHG‐9240A, Shanghai Jinghong Laboratory Equipment). Gross energy for feed, faeces and urine was determined using a bomb calorimeter (6400, PARR Instrument Co.). Ash was measured using a muffle furnace at 550°C for 6 hr with preliminary ashing in an electric heating panel (F47910‐33, Thermo Scientific). The contents of NDF (the solution for concentrate added alpha‐amylase and sodium sulphite, before analysing, immersed in acetone for 2 hr and then air dried; and solution for corn straw and alfalfa added sodium sulphite) and ADF, expressed inclusive of residual ash, were measured using a fibre analyser (2000, ANKOM). Nitrogen content was determined by the Kjeldahl method with copper sulphate and potassium sulphate (1:10, w/w) as a catalyst (UDK159, VELP). Ether extract was measured by weight loss based on DM upon extraction with petroleum ether in an extractor (XT‐15, ANKOM). The NSC content was calculated by subtracting the CP, EE and NDF contents from the OM content. Phosphorus content was determined using a spectrophotometer (Cary 60, Agilent).

#### 2.2.3 Respiration chamber measurement

Six indirect open‐circuit respiration chambers were used with 1 sheep housed per chamber. Methane and CO_2_ production and O_2_ consumption for each ewe were reported as the 2 d average values for individual ewe. The respiration chambers were made with plexiglass walls fitted in steel frames and mounted in a plastic leaky floor with two tubes for gas inlet and outlet. The total volume of 4.86 m^3^ (1.98 m length, 1.46 m width, and 1.68 m height) was ventilated by suction pumps allowing a slight negative pressure within the chambers. The flow rate for each chamber was measured using thermal mass flow metres (GFM57, Aalborg). The flow rates were set at a range from 6 to 10 Nm^3^/h, which gave concentrations of O_2_, CO_2_ and CH_4_ in the air samples within the appropriate measurement range recommended by the manufacturer. Temperature and humidity were set at the range of 15–20°C and 30 ± 10% relative humidity, respectively. Methane, O_2_ and CO_2_ concentrations for atmospheric air entering and exhaust gas leaving each individual chamber through a single port channel were determined by a gas analyser (VA‐3000, Horiba, Kyoto, Japan) on a rotational basis in 21 min intervals (3 min for each chamber and/or ambient air), and the gas was filtered through 3 filtrating apparatuses to ensure that particles of number no more than 5 μm entered the gas analyser. Before the start of each period of respiration measurement, the gas analyser was calibrated using gases with known CH_4_, O_2_ and CO_2_ concentrations and oxygen‐free N_2_ (Dalian Special gases). The CH_4_, CO_2_ and O_2_ concentrations in air samples were determined in the absolute range of 0–200 μl/L, 0–2,000 μl/L and 0%–25% (v/v), respectively. Individual chambers were also calibrated before and immediately after the experiment by releasing known quantities of CH_4_, CO_2_ and O_2_ (analytical grade) into the chambers, and then the recovery rates of CH_4_, CO_2_ and O_2_ were measured. The recovery rates were in the range of 100 ± 2%. Finally, CO_2_ and CH_4_ productions and O_2_ consumption were calculated by multiplying the flow rates by differences in the concentrations in the air samples before into and out of each individual chamber. Heat production (HP) was calculated based on measurements of consumed O_2_ (L/d), produced CO_2_ and CH_4_ (L/d) and urinary N excretion (g/d) (Brouwer, 1965), and CH_4_ energy output (MJ/d) was calculated by multiplying CH_4_ (L/d) by 0.03954 MJ/L.

### 2.3 Statistical analysis

Data were analysed as a 3 × 3 Latin square design using analysis of variance with diet treatment as treatments, experimental periods as rows and serial number of ewes as columns in GenStat statistical software (19th edition, VSN International). Significant differences were declared at *p* ≤ .05, and tendencies were discussed at 0.05 < *p*≤ .1. Differences between means were tested using Fisher's multiple comparisons.

## 3 RESULTS

### 3.1 Nutrient intake and tract apparent digestibility

The effects of alfalfa substitution for concentrate levels on the BW, nutrient intake and digestibility are presented in Table 2. Increasing alfalfa supplementation levels significantly reduced N, EE and P intake, increased roughage, ADF and NSC intake and had no effect on BW, DM, OM and NDF intake and intake capacity (total DM intake/BW). Except for the NSC digestibility, the nutrient digestibility was significantly decreased with an increase in the level of alfalfa substitution for concentrate, especially between the extreme treatments (AH0 and AH30).

**Table 2:** Effects of levels of alfalfa as a substitute for concentrate on BW nutrition intake and digestibility

Items	AH0	AH15	AH30	*SEM*	*p* value
BW and intake, kg or g/d					
BW	32.0	32.6	32.2	0.46	.640
Roughage DM intake	472a	616b	739c	21.9	<.001
DM intake	828	845	837	21.7	.848
DM intake/BW, g/kg	26.1	26.1	26.2	0.63	.998
OM intake	750	762	747	19.5	.857
NSC intake	133a	155b	150b	3.6	<.001
NDF intake	474	473	475	15.3	.995
ADF intake	252a	288b	318c	9.3	<.001
N intake	19.7c	18.7b	17.3a	0.30	<.001
EE intake	20.4c	16.6b	14.2a	0.44	<.001
P intake	4.41c	2.60b	1.48a	0.107	<.001
Digestibility, kg/kg or MJ/MJ					
DM	0.607c	0.584b	0.557a	0.0066	<.001
OM	0.626b	0.600b	0.571a	0.0103	<.001
Digestible OM in total DM	0.567c	0.541b	0.510a	0.0093	<.001
NSC	0.684	0.736	0.735	0.0360	.510
NDF	0.597c	0.552b	0.517a	0.0123	<.001
ADF	0.571b	0.537ab	0.509a	0.0160	.022
N	0.633b	0.569ab	0.541a	0.0240	.038
EE	0.830b	0.761b	0.574a	0.0369	<.001
P	0.523b	0.452b	0.199a	0.0494	<.001

Note

a, b and c: means within the same row and with the same letters are not significantly different (*p* > .05).

### 3.2 Energy utilisation

The effects of alfalfa supplementation levels on energy utilisation are shown in Table 3. An increase in the levels of alfalfa substitution for concentrate had no effects on GE intake, urine energy output, HP and energy balance (EB); significantly decreased DE intake and ME intake; and increased faecal energy output. The energy output as methane for AH0 was significantly greater than that for AH30. Digestible and metabolic energy intakes expressed as a proportion of GE intake were significantly reduced with increasing alfalfa input, and other variables for energy utilisation efficiency were not affected.

**Table 3:** Effects of levels of alfalfa as a substitute for concentrate on energy utilisation

Items	AH0	AH15	AH30	*SEM*	*p* value
Energy intake and output, MJ/d					
GE intake	15.0	15.1	14.7	0.38	.800
Faecal E output	5.17a	5.64ab	6.03b	0.198	.021
Urine E output	0.327	0.347	0.334	0.0149	.623
CH_4_‐E output	0.946b	0.901ab	0.839a	0.0340	.109
DE intake	9.79b	9.42b	8.69a	0.228	.009
ME intake	8.52b	8.17b	7.51a	0.198	.006
HP	6.74	6.36	6.29	0.169	.154
EB	1.78	1.81	1.23	0.221	.135
Energy utilisation, MJ/MJ					
DE/GE	0.654c	0.627b	0.592a	0.0070	<.001
ME/GE	0.568c	0.543b	0.512a	0.0067	<.001
ME/DE	0.869	0.866	0.865	0.0033	.687
HP/GEI	0.451	0.425	0.438	0.0143	.453
EB/GEI	0.117	0.117	0.0742	0.01511	.092
HP/MEI	0.795	0.786	0.852	0.0264	.184
EB/MEI	0.205	0.214	0.148	0.0264	.184

Note

a, b and c: means within the same row with the same letters are not significantly different (*p* > .05).

### 3.3 Nitrogen utilisation

The effects of alfalfa substituting for concentrate on nitrogen utilisation are presented in Table 4. Nitrogen intake and urinary N and NH_4_^+^‐N output were significantly decreased with increasing levels of alfalfa substitution for concentrate, and faecal and manure N output and retained N were not affected. As expected, increasing alfalfa input significantly increased faecal N, expressed as a proportion of N intake and manure N output and decreased urinary N/manure N.

**Table 4:** Effect of levels of alfalfa as a substitute for concentrate on nitrogen utilisation

Items	AH0	AH15	AH30	*SEM*	*p* value
N intake and output, g/d					
N intake	19.7c	18.7b	17.3a	0.30	<.001
Faecal N output	7.17	8.16	7.95	0.495	.349
Urinary N output	9.96b	8.93ab	7.79a	0.738	.141
Manure N output	17.1	17.1	15.7	0.93	.495
Retained N	2.57	1.64	1.54	0.913	.683
Urinary NH_4_^+^‐N output	0.146b	0.114a	0.107a	0.0073	.003
N utilisation, g/g					
Faecal N/N intake	0.367a	0.431ab	0.459b	0.0240	.038
Urinary N/N intake	0.507	0.495	0.458	0.0396	.664
Manure N/N intake	0.873	0.927	0.917	0.0465	.696
Retained N/N intake	0.127	0.0734	0.0834	0.0465	.696
Faecal N/manure N	0.423a	0.475ab	0.503b	0.0254	.100
Urinary N/manure N	0.577b	0.525ab	0.497a	0.0254	.100
Urinary N/faecal N	1.44	1.25	1.07	0.136	.176
Urinary NH_4_+‐N/urinary N	0.0158	0.0131	0.0143	0.00121	.309

Note

a, b and c: means within the same row and with the same letters are not significantly different (*p* > .05).

### 3.4 Methane emissions

The effects of alfalfa supplementation levels on methane emissions are presented in Table 5. An increase in the alfalfa supplementation levels significantly decreased CH_4_ emissions expressed as a proportion of BW and of the DM, OM, GE and ME intake. Other CH_4_ emissions variables were not affected, but methane emissions per day for AH0 was significantly greater than that for AH30.

**Table 5:** Effects of levels of alfalfa as a substitute for concentrate on methane emissions

Items	AH0	AH15	AH30	*SEM*	*p* value
CH_4_ emissions, g/d	17.1b	16.3ab	15.2a	0.62	.109
CH_4_/DM intake, g/kg	20.8b	19.4ab	18.1a	0.55	.010
CH_4_/OM intake, g/kg	22.9b	21.5ab	20.3a	0.60	.021
CH_4_/digestible DM intake, g/kg	34.2	33.2	32.6	0.93	.475
CH_4_/digestible OM intake, g/kg	36.6	35.9	35.6	0.98	.771
CH_4_/BW, g/kg	0.540b	0.505ab	0.473a	0.0245	.043
CH_4_‐E/GE intake, MJ/MJ	0.0635b	0.0602ab	0.0569a	0.00168	.039
CH_4_‐E/DE intake, MJ/MJ	0.0971	0.0960	0.0964	0.00260	.960
CH_4_‐E/ME intake, MJ/MJ	0.112	0.111	0.112	0.0034	.814

Note

a, b and c: means within the same row and with the same letters are not significantly different (*p* > .05).

## 4 DISCUSSION

### 4.1 Intake and tract apparent digestibility

In present study, the DM intake was not affected by an increase in the levels of alfalfa substitution for concentrate, which was in agreement with the results of Hales, Brown‐Brandl, and Freetly (2014), who reported that DM intake was not significantly different when alfalfa was substituted at various mixing ratios for concentrate‐based diets. Ramos et al. (2009) reported that shifting forage:concentrate ratio from 70:30 to 30:70 had no effect on the DM intake for sheep fed diets with good‐quality alfalfa or grass hay. These results revealed that the response in total DM intake to high concentrate supplementation levels was weakened or overridden by an increase in forage quality (Blaxter, Wainman, & Wilson, 1961; Keady & Hanrahan, 2015). Dry matter intake was strongly correlated with factors that affected the extent of digestion and the flow rate of digesta through the gastrointestinal tract (Keady & Hanrahan, 2015), which could be evaluated by NDF concentration. The NDF concentration, which is the primary factor restricting DM intake and digestibility (McDonald et al., 2011), determined the degree of rumen fill and the passage rate through rumen (NRC, 2007). In this study, the NDF content was not affected by an increase in alfalfa input level (571, 558 and 562 g/kg for AH0, AH15 and AH30, respectively, *p* > .1), which might be the main reason for the similar DM intake between the benchmark and alfalfa diets. Meanwhile, compared with concentrate, the longer particles of alfalfa increased the rumination and salivation of animals, which also contributed to similar DM intake (Carvalho et al., 2019).

In this study, the digestibility was significantly decreased by an increase in the levels of alfalfa substitution for concentrate. This reduction might be partly attributed to legumes containing a higher ADF content (304, 340 and 377 g/kg DM ADF contents for AH0, AH15 and AH30, respectively, *p* < .001) than concentrate and the fibre in forage having a greater inhibitory effect on the rate of digestion. Lower nutrient digestibility for AH15 and AH30 was also partly caused by alfalfa containing anti‐nutritional saponins and tannins that reduced the rumen protozoa concentration (Morgavi et al., 2011; Owens, Provenza, Wiedmeier, & Villalba, 2012; Sen et al., 1998), which also contributed to lower fibre and N digestibility.

### 4.2 Nitrogen utilisation

This result showed that N intake and urinary N and NH_4_^+^‐N output were decreased with an increase in alfalfa input levels. In the current study, N excretion was shifted from urine to faeces after the decrease in N intake was caused by an increase in alfalfa input, which was in line with the urinary N excretion being lower in high‐NSC diets (Kebreab et al., 2010). Reducing N intake could be manipulated as an effective strategy to decrease N excretion and improve N intake partitioning, as reported by Yan, Frost, Keady, Agnew, and Mayne (2007), who observed that an increase in dietary N concentration by 1 g/kg of DM could increase 7.6 g/kg urinary N output/N intake for beef cattle. As demonstrated by Silva, Sobrinho, Trindade, Resende, and Bakke (2004), the CP level could be reduced to 130 g/kg DM, but when lower than 11%, the CP level probably could not support optimal microbial growth (Fanchone et al., 2013; Pathak, 2008; Silva et al., 2004). In present study, ammonia production was reduced by 26.9% after CP content was reduced from 149 to 131 g/kg DM, and the reduced value was higher than 20% reported by Kebreab, France, Mills, Allison, and Dijkstra (2002), who observed the reduction of CP concentration reaching approximately 160 g/kg DM for cows fed 30 different diet types consisting of 10 grass silages and 6 concentrates. This result implied that a reduction of N intake manipulated by alfalfa input to replace concentrate could shift N excretion from urine to faeces and could reduce the urinary N and ammonia output to mitigate N pollution.

In the present study, retained N, expressed as a proportion of N intake, was not affected when the level of alfalfa substitution for concentrate increased. This result might be due to the increase in the retained N:N intake caused by increased ME content (10.26, 9.72 and 9.00 MJ/kg DM ME concentration for AH0, AH15 and AH30, respectively, *p* < .001), which was probably offset by an increase of N intake and similar digestible OM intake: N intake (23.9, 24.5 and 24.6 g/g digestible OM intake: N intake for AH0, AH15 and AH30, respectively, *p* > .1). Furthermore, the lower microbial protein yield in poor‐quality forage‐based diets could be compensated by improving basal forage quality rather than increasing the amount of concentrate input (Pathak, 2008), and lower N losses and improved N utilisation efficiency are probably achievable by using a mixture of energy sources from low rate of degradability and well‐balanced diets (Kebreab et al., 2010). Alfalfa containing higher readily degradable fraction of protein than cereal grains (Pathak, 2008) could optimise synchronisation between N and carbohydrates degradation, which was more important than the CP level (Milis & Liamadis, 2008). Meanwhile, alfalfa containing saponin and tannins increases the efficiency of microbial protein synthesis because of a decrease in protozoa concentration (Morgavi et al., 2011; Sen et al., 1998). Compared with AH0, the synchronisation rate for carbohydrate degradation and N release for AH15 and AH30 was improved by a high readily degradable N and low rate of N and energy release for alfalfa, and greater availability of carbohydrates (16.2, 18.5 and 18.3 g/kg DM NSC content for AH0, AH15 and AH30, respectively, *p* < .01). These aspects contributed to that AH15 and AH30 diets could sustain no significant difference in the N retained with shifting N excretion from urine to faeces, and the ammonia output was decreased.

### 4.3 Methane emissions

Total DM intake is the critical driver of CH_4_ production per day (Ellis, Odongo, McBride, Okine, & France, 2007; Yan et al., 2010; Zhao, Aubry, Annett, O’Connell, & Yan, 2016), which is also directly related to diet quality (Boadi & Wittenberg, 2002). An increase of 0.05 g/d CH_4_ production was caused by each 1 g digested NDF intake increased (Santoso, Mwenya, Sar, & Takahashi, 2007), and the relative value in this study was 0.034 g/d. The significantly lower digested NDF intake (284b, 262ab and 247a g/d for AH0, AH15 and AH30, respectively) for AH30 contributed to significantly lower CH_4_ production (g/d), compared with AH0.

Methane emissions per unit of intake was determined by fermented carbohydrates and levels of intake (Johnson & Johnson, 1995), which was negatively related to concentrate levels, forage quality and available energy content (Yan et al., 2010; Zhao et al., 2016). A decline of 3.03 g CH_4_/kg DM intake occurred for each multiple in ME intake beyond ME_m_ (Yan et al., 2010), which was not observed in present study. This reflected the high inconsistency of response, possibly due to interactions of concentrate input levels with other nutrient components and roughage quality of diets. The reduction was observed when concentrate level was beyond 50% (Islam, Abe, Hayashi, & Terada, 2000), as well as when intake levels were no less than 2.5 times the requirement for maintenance (Morgavi et al., 2011). As Jiao et al. (2014) reported, the CH_4_/DM intake (g/kg) was not affected after concentrate added from 2 to 4 kg/d and significantly decreased after concentrate added to 6 kg/d, and Boadi, Wittenberg, and McCaughey (2002) showed that grazing season rather than concentrate input had an effect on the CH_4_ yield for steers grazed on alfalfa and meadow bromegrass pastures supplemented barley grain. In addition to the concentrate level and the level of feeding, forage type also had an effect on CH_4_ yield. Legumes rich in secondary metabolites such as saponins and tannins (Morgavi et al., 2011) had lower methanogenic potential because of the reduced concentrations of rumen protozoa and methanogens. Methane production expressed as the proportion of DM and GE intake was positively correlated with nutrient digestibility, especially digestion of NDF and ADF (Santoso et al., 2007; Stergiadis et al., 2016). In present study, CH_4_ production per kg of DM or MJ GE intake was significantly decreased by an increase in the levels of alfalfa as a substitute for concentrate, possibly because the increase in alfalfa input overrode the effect of the concentrate levels (424, 267 and 114 g/kg DM for AH0, AH15 and AH30, respectively, *p* < .001) and feed level (1.86, 1.73 and 1.58 ME_m_ for AH0, AH15 and AH30, respectively, *p* < .001), and this reduction was attributed to the significant decrease in nutrient digestibility and the lower methanogenic potential of the alfalfa.

In this study, CH_4_ production as a proportion of GE intake was 6.35%, 6.02% and 5.69% for AH0, AH15 and AH30, respectively, with a mean value of 6.02%. The present mean value is lower than that (8.0%) reported by McDonald et al. (2011) but close to the recommendation (6.5%) of IPCC (2006) for estimating CH_4_ production when CH_4_ emissions data are not available. This result clearly states that using the default values recommended by McDonald et al. (2011) and/or IPCC (2006) probably causes a certain range of error for predicting CH_4_ emissions from ewes offered diets with corn straw and alfalfa as forage. Further study for ewes fed improved quality roughage and/or diets with legumes rich in saponins and tannins as forage is necessary to quantify CH_4_ emissions, and exploration is required to determine whether the decrease in ruminant production caused by lower nutrient digestibility and energy utilisation obtained from alfalfa used to replace a portion of concentrate diet could be compensated by lower CH_4_ and urinary N and NH_4_^+^‐N output and by lower feed cost.

## 5 CONCLUSION

The present study evaluated the effect of replacement rates of alfalfa hay for concentrates (0%, 15% and 30% in total diets (DM basis), respectively) in sheep offered diets containing 60% corn straw and 40% concentrates/alfalfa hay. The results demonstrated that, in comparison with sheep given the benchmark diet, the sheep offered alfalfa diets could sustain similar DM intake and energy and N retention, although alfalfa diets had significantly lower DM, NDF, N and energy digestibility. Diets including alfalfa hay had no significant effects on HP or energy retention over ME intake or on urine N or manure N over N intake, whereas increasing alfalfa inclusion rates significantly decreased CH_4_/DM intake, urinary N and NH_4_^+^‐N output per day. The present study indicates that alfalfa hay could be used to replace a certain level of concentrate for adult sheep with few negative effects on feed intake, N or energy retention or CH_4_ emissions. This result needs to be validated in long‐term studies.

The results provide new information and recommendations for farmers who engage in sheep raising to use alfalfa to replace a portion of the concentrate supplement for more sustainable sheep production in the region, and they also provide support for the implementation of the “grain to forage” policy.

## 6 ANIMAL WELFARE STATEMENT

All animal management and experimental procedures for this study were approved by the Animal Ethics Committee of Lanzhou University and conducted under the rules and regulations of experimental field management protocols (file No. 2010‐1 and 2010‐2) in accordance with the Guides for Management of Laboratory Animals in Gansu Province, China (Gansu Provincial Department of Science & Technology, 2005).
